# Cardiac Involvement and Arrhythmias Associated with Myotonic Dystrophy

**DOI:** 10.31083/j.rcm2304126

**Published:** 2022-04-02

**Authors:** Daniel McBride, Amrish Deshmukh, Supriya Shore, Melissa A. Elafros, Jackson J. Liang

**Affiliations:** ^1^Electrophysiology Section, Division of Cardiology, Ann Arbor, MI 48109, USA; ^2^Heart Failure Section, Division of Cardiology, University of Michigan, Ann Arbor, MI 48109, USA; ^3^Neuromuscular Section, Division of Neurology, University of Michigan, Ann Arbor, MI 48109, USA

**Keywords:** myotonic dystrophy, nucleotide expansion, heart failure, arrhythmia, ventricular tachycardia, sudden death

## Abstract

Myotonic dystrophy is an autosomal dominant genetic disease of nucleotide expansion resulting in neuromuscular disease with two distinct subtypes. There are significant systemic manifestations of this condition including progressive muscular decline, neurologic abnormalities, and cardiac disease. Given the higher prevalence of cardiac dysfunction compared to the general population, there is significant interest in early diagnosis and prevention of cardiac morbidity and mortality. Cardiac dysfunction has an origin in abnormal and unstable nucleotide repeats in the *DMPK* and *CNBP* genes which have downstream effects leading to an increased propensity for arrhythmias and left ventricular systolic dysfunction. Current screening paradigms involve the use of routine screening electrocardiograms, ambulatory electrocardiographic monitors, and cardiac imaging to stratify risk and suggest further invasive evaluation. The most common cardiac abnormality is atrial arrhythmia, however there is significant mortality in this population from high-degree atrioventricular block and ventricular arrhythmia. In this review, we describe the cardiac manifestations of myotonic dystrophy with an emphasis on arrhythmia which is the second most common cause of death in this population after respiratory failure.

## 1. Introduction

Myotonic dystrophy is an autosomal dominant condition and represents the most common inherited neuromuscular disease in adults. It is frequently associated with cardiac complications which run the gamut from asymptomatic first-degree atrioventricular block to ventricular fibrillation and sudden cardiac death.

The condition is caused by unstable, simple nucleotide tandem repeats in the dystrophia myotonica protein kinase (*DMPK*) gene for myotonic dystrophy type 1 (DM1) and in the cellular nucleic acid binding protein gene (*CNBP*) for myotonic dystrophy type 2 (DM2) [1, 2]. In DM1, variations in organ-specific phenotypes, such as arrhythmia, are thought to be dependent on the length of the unstable nucleotide repeats in progenitor cells [3]. The same relationship is not established in DM2, which has a milder phenotype [4].

In general, the clinical course of DM1, and to a lesser extent DM2, involves the insidious development of skeletal muscle weakness with wasting and myotonia. The most common systemic manifestations of the disorder are respiratory and cardiac disturbances. Other manifestations include ophthalmopathies (premature cataracts), endocrinopathies (diabetes mellitus) and disorders of the alimentary system [5].

## 2. Pathophysiology and Genetics

Myotonic dystrophy has an incidence of 1/8000 live births and a worldwide prevalence of 2.1–14.3/100,000 inhabitants [6]. Geographically, there are variations in prevalence of between 2.2–5.5/100,000 in Western Europe, however the disease is rarer in South-East Asian and African populations [7, 8]. In the United States, blood-spot testing in newborns completed in New York suggests a prevalence of 4.8/10,000 [9]. These figures imply that the true prevalence is likely underestimated, and the condition is underdiagnosed likely due to under-recognition.

**Fig. 1.**
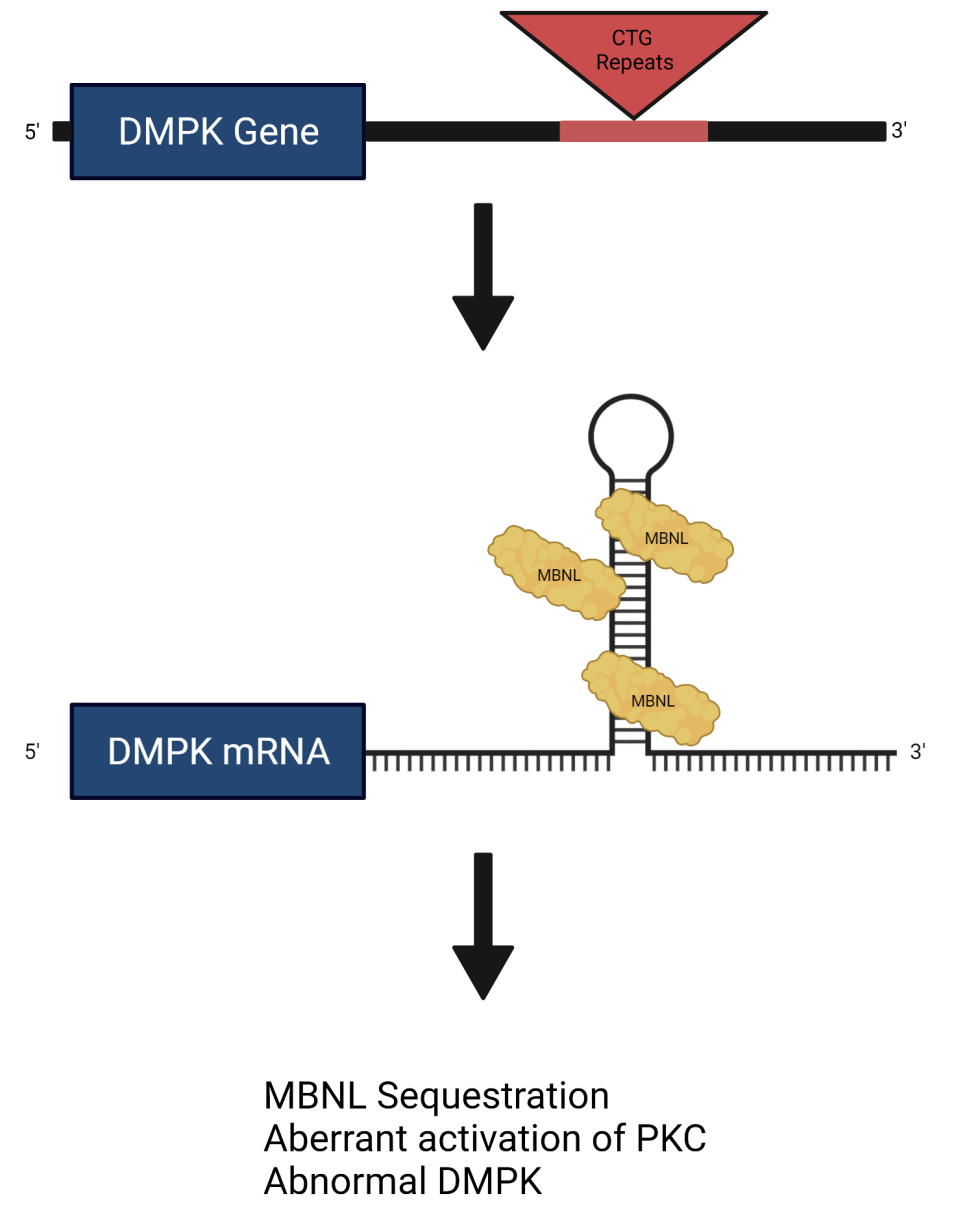
**Graphic representation of CTG-repeat effect leading to transcription of mutant RNA which accumulate in cell nuclei and ultimately promote abnormal splicing of proteins**. Muscleblind-like (MBNL) protein splice abnormal mRNA and co-localize to the nucleus resulting in functional sequestration and abnormal activation of protein-kinase C (PKC). Created with BioRender.com.

DM1 results from unstable trinucleotide repeats of the sequence cytosine-thymine-guanine (CTG) in the 3’ untranslated region of the *DMPK* gene, on chromosome 19q 13.3 [1]. This causes the transcription of mutant ribonucleic acids (RNA) which accumulate in cellular nuclei and, in turn, have a toxic effect on specific RNA-binding protein families leading to loss of function [10]. The loss of function of RNA-binding proteins directly causes abnormal splicing of several genes leading to clinical manifestations of DM1 that vary based on tissue and trinucleotide repeat length. Genes affected include the bridging integrator 1 gene, the cardiac troponin T gene, the cardiac sodium channel Nav 1.5 gene, the *NKX 2-5* gene, the insulin receptor gene, and the skeletal muscular chloride gene [11, 12, 13, 14, 15, 16]. The various effects of these genes and a graphical representation of this process are listed in Table 1 (Ref. [11, 12, 13, 14, 15, 16]) and Fig. 1, respectively.

**Table 1.** **Gene mutations resulting from DM due to RNA-binding protein sequestration and subsequent abnormal splicing**.

Gene	Downstream effect
Bridging Integrate 1 (*BIN1*)	Translation of abnormal T-Tubules causing impaired excitation-contraction and muscle weakness [11]
Cardiac Troponin T (*TNNT2*)	Disruption of striated muscle cells [12]
Insulin Receptor	Insulin resistance [15]
Skeletal Muscular Chloride Channel	Myotonia [16]
Cardiac Sodium Channel Nav 1.5 (*SCN5A*)	Arrhythmia [13]
Cardiac transcription factor (*NKX2-5*)	Arrhythmia [14]

DM2 has a similar pathophysiology; it results from the tetranucleotide repeat of cytosine-cytosine-thymine-guanine (CCTG) in intron 1 of the *CNBP* gene on chromosome 3q 21.3 [2]. Although *CNBP* and *DMPK* genes encode for unrelated proteins, the similar pathophysiology support the toxic effect of abnormal RNA transcripts [10, 17].

Phenotypic heterogeneity exists for DM1 which modestly correlates with number of trinucleotide repeats as well as level of somatic mosaicism that is seen with this disease process [3]. Somatic mosaicism, the idea that genetically separate populations of cells can coexist within an individual, is likely due to tissue-specific affinity for increased repeat expansion. This has been demonstrated in newborn populations, where CTG repeats are more expanded in the heart and muscle tissue as compared to saliva or peripheral white blood cells [18]. Genetic anticipation is also observed in DM1, as subsequent generations are more likely to develop severe forms of the disease vis-à-vis higher numbers of trinucleotide repeats [19]. It is also suggested that when females transmit the dominant gene to progeny, there is a larger range of trinucleotide expansions and therefore high intergenerational mean variation than when comparable males transmit the dominant gene [20].

There is not a well-established relationship between the number of tetranucleotide repeats and disease severity with DM2 as there is for DM1 [4].

## 3. Clinical Characteristics

There are four clinical subtypes of DM1: congenital, childhood-onset, adult-onset, or classical, late onset or mild [21]. Adult-onset is the most common form of DM1 [22]. Clinically, mild, and adult-onset disease are notable for adult-onset myotonia and weakness involving the facial muscles with progression to involve the distal limb muscles. Multi-system organ dysfunction is more prevalent in classical disease [23]. Childhood and congenital disease can be more severe, with weakness and multi-organ system dysfunction notable from utero, with polyhydramnios and hypotonia, to adolescence with early onset respiratory dysfunction and weakness [24, 25, 26]. Based on Dutch registry data, life expectancy in DM1 is estimated at approximately 60 years for both males and females [27]. DM2 has a milder phenotype than DM1 with adult onset myotonia, proximal muscle pain, weakness, and cataracts.

The condition previously relied on clinical exam and EMG with possible muscle biopsy for diagnosis, however genetic testing has emerged as the present gold standard [21, 23].

A general classification of clinical spectrum for DM1 has been suggested based on number of trinucleotide repeats, with higher numbers causing earlier and more severe disease presentation [28, 29]. The subtypes include mild (50–150 repeats), classical (50–1000 repeats), childhood onset (>800 repeats) and congenital (>1000 repeats) [23].

## 4. Cardiac Manifestations

Cardiac abnormalities including cardiomyopathy, conduction disturbances, and arrhythmias in patients with myotonic dystrophy are common and increase with age after diagnosis. Cardiac involvement impacts up to 80% of patients with DM1 and 20% of patients with DM2 and results from progressive myocardial fibrosis which causes a dilated cardiomyopathy and conduction system abnormalities [30, 31, 32]. A nationwide Danish cohort study followed 1146 patients with either clinical or genetically proven myotonic dystrophy and found a standardized incidence ratio of 3.42 [95% CI 3.01–3.86] for any cardiac disease [33]. A cross-sectional study in Utah found that the relative risk of any cardiac conduction disorder was 60 times that of the normal population (95% CI 29.9–108.6) [34].

Given the prevalence of cardiac involvement in patients with DM, there is organizational focus via the AHA and Myotonic Dystrophy Foundation on the research, screening, and development of cardiac-specific therapeutics. In the next sections we will briefly summarize the current understanding of cardiomyopathy and arrhythmia in this patient population with a focus on arrhythmia.

## 5. Arrhythmia

Arrhythmia is the second most-common cause of death in patients with DM as identified in the largest prospective cohort to-date [35]. One mechanism of arrhythmia is the upregulation of *NKX2-5* gene and abnormal splicing of the *SCN5A* gene encoding the cardiac sodium channel NAv1.5. These mutations can cause or contribute to delayed atrio-ventricular conduction, interventricular conduction delay and a host of atrial and ventricular arrhythmias [13, 14]. Dominant *NKX2-5* mutations are associated with congenital atrial septal defects, heart block and atrial fibrillation [36]. Loss of function mutations seen in the *SCN5A* gene are akin to other conduction syndromes such as long QT3 syndrome and Brugada syndrome [37].

Ventricular fibrillation (VF) may occur as the result of unstable or untreated ventricular tachycardia (VT) or may arise de novo in a similar mechanism to Brugada Syndrome owing to *SCN5A* mutation [38]. Mouse models haves shown that alternative splicing of *SCN5A* via a mechanism akin to DM promotes arrhythmia and conduction delay typical of DM. In such models, there is a high burden of PR-interval prolongation and sudden death [38]. Further studies using optical mapping analysis have revealed slower conduction velocities and longer action potential durations and restitutions. These conduction system abnormalities allow for a greater chance of reentrant tachycardias and the induction of VT/VF with pacing compared to wild-type controls [39].

Another mechanism of arrhythmia is thought to be related to disease-specific anatomic changes throughout the conduction system. Post-mortem evaluation of cardiac tissue from one series of 12 patients with DM has revealed fibrosis, fatty infiltration, lymphocytic infiltration and atrophy in the sinoatrial (SA)-nodal tissue, atrioventricular (AV)-nodal tissue and throughout the AV bundle consistent with antemortem cardiac diagnoses [30]. Anatomical changes in the nodal tissues of both the SA node and AV node contribute to sick sinus syndrome and varying degrees of AV-block in patients with DM [40]. Posited mechanisms for ventricular arrhythmia include re-entrant VT due to anatomical abnormalities in addition to a predisposition for bundle branch re-entry and fascicular VT resulting from disease within the His-Purkinje system [41, 42]. Such conduction abnormalities are posited to represent a significant contributor to arrhythmic mortality in patients with DM [43].

**Fig. 2.**
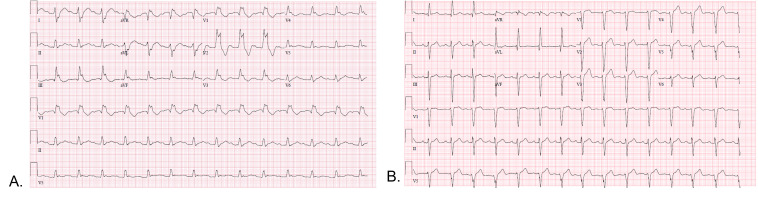
**ECGs obtained from patients with DM type 1**. ECG “A” shows sinus rhythm with first degree atrioventricular delay and right bundle branch block. ECG “B” shows sinus rhythm with non-specific interventricular conduction delay.

Clinically, these abnormalities manifest on the electrocardiogram (ECG)- often prior to the development of cardiac symptoms, and may precede muscular symptoms [44]. ECG abnormalities are present in approximately 65% of DM1 and 20% of DM2 patients. In DM1 patients, first degree atrioventricular delay is the most common abnormality (42%), followed by non-specific intraventricular conduction delay (12%) [35]. Other abnormalities reported in patients include left- or right- bundle branch block, pathologic q-waves and repolarization abnormalities [45]. A representative example is seen in Fig. 2. In a study of 94 consecutive genetically confirmed DM1 and DM2, DM1 patients were found to have a larger incidence of intraventricular and atrioventricular conduction defects though DM2 had a larger incidence of nonsustained supraventricular and ventricular tachycardias [46].

## 6. Atrial Arrhythmias

Atrial arrhythmias are the most common clinical arrhythmias associated with DM1 and represent an independent predictor of increased mortality [35]. Atrial fibrillation and atrial flutter have an estimated prevalence of 10.9%. and 8.5%, respectively in DM1 [47, 48]. Management of atrial arrhythmias in this population is the same as current standard-of-care practices. This includes anticoagulation for stroke prevention guided by CHA2DS2VASc score, rate, and rhythm control. Owing to AV dysfunction, rapid ventricular rate may be less common in this population.

Notably, the DM population has higher incidence of risk factors for the development of atrial arrhythmia, including obstructive sleep apnea and metabolic syndrome [49, 50]. As in non-DM patients, it is critical to manage these risk factors to potentially reduce arrhythmia burden. Additional patient-centered factors, such as immobility or fall frequency should be considered when treating this population. Therefore, a higher clinical index of suspicion for atrial arrhythmia is recommended based on routine ECG surveillance and the presence of clinical symptoms [51].

Ambulatory monitoring for patients with DM has been suggested by the AHA as a means of detecting arrhythmia. In retrospective observational data, annual 24-hour holter monitoring was not useful for predication of cardiovascular events [52]. However, there is no comparable data with longer-term event monitors, which have shown increased ability to detect atrial arrhythmias after stroke [53].

Treatment with rate and rhythm control medications may present a challenge if there is underlying sino-atrial or atrio-ventricular conduction abnormalities and warrants close monitoring. Ablation for atrial arrhythmias is known to be effective in patients with DM [54]. The ideal ablation approach beyond pulmonary vein isolation (PVI) remains unclear, especially for those with more persistent atrial arrhythmias. Electro-anatomical mapping in these patients has suggested low-voltage zones to be an additional target for ablation beyond PVI. These low-voltage zones in patients with DM have been theorized to be a primary dysfunction related to the anatomic myocardial abnormalities characteristic of DM in contrast to secondary low-voltage zones typical of comorbidities related to atrial fibrillation [55].

## 7. Sino-Atrial and Atrio-Ventricular Nodal Dysfunction 

Proximal conduction abnormalities, defined as those above the AV-node, are less common than those distal to the AV node although both are significantly associated with the presence of inducible atrial arrhythmias. The most common conduction abnormality noted in electrophysiological studies (EPS) of patients with DM is a prolonged H-V interval [56].

Prospective studies evaluating conduction in patients who underwent prophylactic pacemaker implantation identified H-V interval >70 ms to be associated with progression to complete AV dissociation [57]. Non-invasive testing with surface ECG suggests similar findings as ECG abnormalities including PR interval of ≥240 ms, QRS duration ≥120 ms, second, or third-degree AV block were independent predictors of mortality in patients with predominantly type 1 DM followed prospectively [35]. Delayed conduction assessed by these metrics has also been associated with the cumulative risk of sudden death and need for pacemaker implantation [58]. As such, prophylactic pacemaker implantation in patients with these ECG abnormalities is a class IIb recommendation by both the AHA and the European society of cardiology [59, 60]. Subsequent studies using EPS have called into question the predictive value of ECG in assessing for infra-Hisian conduction block due to discordance between the PR interval, QRS duration and the HV interval as measured on EPS [61]. The AHA currently adheres to a class IC recommendation that for any surface ECG abnormality as described above, symptoms of arrhythmia should have annual cardiac examination and be considered for EPS or device implantation [6]. There are retrospective registry data to suggest that patients with abnormal ECG findings who underwent pacemaker implantation had improved survival compared to propensity matched cohorts who did not receive pacemakers [62].

Pacing strategies in DM do not differ from standard of care. As atrial arrhythmias in this population are more common with disease progression, there has been interest in whether atrial pacing may reduce atrial arrhythmia burden. Single center prospective data from 60 patients found that an atrial preference pacing algorithm significantly reduced atrial arrhythmia burden at both 1- and 2-year follow-up compared to standard DDDR pacing modes [63].

Despite implantation of prophylactic pacemaker, there have been incidences of sudden death in patients with DM which suggests unstable ventricular arrhythmia as a likely culprit [35, 64].

## 8. Ventricular Arrhythmias 

Ventricular arrhythmias, including monomorphic ventricular tachycardia (VT), polymorphic VT, and ventricular fibrillation (VF) have been described in patients with DM. Interestingly, ECG abnormalities suggestive of AV conduction disease are also associated with a higher likelihood of ventricular arrhythmia in prospective studies [35, 65, 66]. Furthermore, in a single-center prospective study of 53 patients with myotonic dystrophy and 47 matched controls, signal-averaged electrocardiography findings of late potentials were associated with the development of ventricular arrhythmia at mean follow-up of 31 months [67]. Due to high false positive rates, signaled average ECG receives a IIb/B recommendation from the AHA for monitoring in younger DM patients [6].

VTs involving the conduction system such as bundle-branch reentrant and fascicular VT are amenable to catheter ablation as in other patient populations, although prospective outcomes data in this cohort of DM patients is sparse [41, 42]. Reentrant VT due to anatomical abnormalities is likely to be amenable to ablation, although challenges with mid-myocardial substrate are likely to be present based on CMRI imaging showing a preponderance of delayed gadolinium enhancement in this population [68].

Despite the increased risk of ventricular arrhythmia in this population, there are no guidelines on the implementation of implantable cardioverter-defibrillator (ICD) devices in these patients unless criteria are met for primary prevention due to left-ventricular systolic dysfunction or for ventricular arrhythmia inducibility during EPS. The Heart Rhythm Society will soon release updated guidelines for risk stratifying these patients. Current AHA recommendations rely on close monitoring of surface ECG, event monitoring and echocardiography in conjunction with clinical symptoms to refer for electrophysiologic evaluation as summarized in Table 2 (Ref. [6]). In our practice, we perform EPS with programmed ventricular stimulation to risk stratify patients with DM and high risk features (i.e., history of syncope, reduced LVEF, delayed gadolinium enhancement on MRI, documented nonsustained VT, *etc*.) and implant ICD in those who are inducible for sustained VT or VF based on consensus guidelines for other inherited cardiomyopathies and channelopathies such as in patients with Lamin A/C mutation or Brugada syndrome [69]. Importantly, in cases where sustained VT/VF are not inducible and the decision is made to not implant ICD, implantable loop recorders can be helpful to monitor for subsequent development of sustained ventricular arrhythmias due to uncertain cardiac progression over time. Furthermore, among DM patients in whom pacemaker implantation is recommended for bradycardia or AV block, implantation of ICD rather than pacemaker should be considered if concordant with the patient’s long-term goals. VT catheter ablation has been successful in patients with myotonic dystrophy, particularly in patients with classical outflow-tract and bundle-branch reentrant inducible VT on EPS [70, 71]. There is little data regarding treatment efficacy of antiarrhythmic medications specifically for these patients, however it should be noted that off target effects of drugs with multiple ion-channel targets, such as amiodarone, may worsen sinus and atrioventricular node dysfunction if it is present.

**Table 2.** **Summary of current American Heart Association guidelines [6]. Class I designates a strong recommendation, Class 2a is a moderate recommendation, Class 2b is a weak recommendation, Class 3 is a weak recommendation with no benefit noted. Level of evidence A reflects high-quality evidence, Level of evidence B reflects moderate quality evidence and may be randomized or non-randomized, Level of evidence C is limited evidence and may rely on expert opinion**.

Recommendation	Size of treatment effect
At the time of diagnosis of DM, recommend cardiology evaluation: ECG, Echocardiogram, Ambulatory Rhythm Monitoring	Class I, Level of Evidence C
Patient with arrhythmic symptoms or ECG showing non-sinus rhythm, PR >240 ms, QRS >120 ms or evidence of atrioventricular block should be considered for annual evaluation, EPS, or device implantation	Class I, Level of Evidence C
DM patients with normal left ventricular systolic function who lack symptoms or abnormal ECG may be reasonably followed with annual exam, ECG, event monitoring and by echocardiography every 2 to 4 years	Class IIa, Level of Evidence B
Young DM1 patients should undergo serial exercise stress testing and signal-averaged ECG	Class IIb, Level of Evidence B

## 9. Cardiomyopathy/Structural Heart Disease

CTG nucleotide expansion in myocytes leads to hairpin RNA structures that bind splicing factors in cellular nuclei resulting in abnormal splicing of the *TNNT2* and *SCN5A* genes which, in turn, is thought to contribute to cardiomyopathy [12]. Cardiomyopathy associated with DM1 and DM2 progresses with age; as such regular echocardiography with strain imaging is a class IC recommendation by the American Heart Association (AHA) to track progression [6, 51]. Echocardiogram is recommended at the time of diagnosis and then every 2–4 years if the initial echocardiogram is within normal limits [6].

Echocardiography results from 100 DM patients via a multicenter data registry revealed the most common echocardiographic findings are left-ventricular hypertrophy (LVH) (19.8%), left-ventricular dilatation (18.6%) and decreased left ventricular systolic function (14%) [72]. More recently, a study of echocardiography with global longitudinal strain (GLS) in a prospective cohort of forty-six DM1 patients found abnormal GLS to be highly predictive of cardiovascular events independent of systolic function [73]. Interestingly, clinical heart failure symptoms were only present in 1.8% of patients as muscular weakness and fatigue can mask typical symptoms [72].

Cardiac magnetic resonance imaging (CMR) can identify fatty infiltration and early signs of fibrosis through delayed gadolinium enhancement (LGE) and may be beneficial for early diagnosis of cardiac involvement in the absence of electrocardiographic or echocardiographic findings [74]. The most common pattern of delayed gadolinium enhancement in one study showed mid-myocardial enhancement in the basal inferolateral wall [75]. Another study of fifty-seven DM1 patients who underwent CMR found myocardial abnormalities as an independent risk factor for the occurrence of atrial fibrillation and atrial flutter [76]. CMR in DM2 has shown subepicardial LGE in the basal inferolateral wall and is also predictive of arrhythmia [77].

Management of cardiomyopathy in DM centers on the use of guideline-directed medical therapies (GDMT) and cardiac resynchronization therapy as recommended for stage C heart failure with reduced ejection fraction. Data to suggest slowed progression of left-ventricular dysfunction with GDMT and cardiac resynchronization is abstracted from treatment response to these therapies seen in other muscular dystrophies [78, 79].

There are case reports of cardiac transplantation in patients with DM1 and DM2, with short-term and long-term data still under collection. Reasonably, there is concern with transplantation in this population given difficulties with extubation and rehabilitation due to muscle weakness [80].

## 10. Disease-Specific Treatments for Myotonic Dystrophy

In general, specific therapies for myotonic dystrophy are aimed at addressing the organ system involved. Due to muscle involvement, exercise therapies have been postulated to be of benefit, although there have been no studies to date to show improved outcomes [81]. In patients with congenital or childhood DM, exercise was noted to induce ventricular arrhythmia and therefore ECG monitoring during exercise may be of benefit [25].

Pharmacologic therapies for myotonia and muscle weakness have traditionally targeted the sodium channels on skeletal muscle with sodium channel blockers. Importantly, medications used for this condition including mexiletine, flecainide and propafenone can worsen conduction abnormalities, which may be an issue in patients without pacemakers [82, 83, 84, 85, 86].

Due to inactivity and impaired glucose tolerance, patients with DM are at increased risk for atherosclerotic vascular disease [87]. While the widespread use of statin conveys a low risk of myopathies, discontinuation of therapy may be more common in the DM population due to baseline myopathic symptoms. Current recommendations suggest serial creatine kinase measurement in this population to help distinguish true myopathy [88].

Future therapeutics may focus on the applications of antisense oligonucleotides to combat the intranuclear toxic RNA effect to diminish the burden of abnormally spliced genes [89]. Gene therapy has been proposed as a feasible option to eliminate nucleotide expansion and is under investigation [90].

## 11. Conclusions

The clinical manifestations of DM are complex yet are becoming increasingly treatable with modern therapies. Current cardiac-specific therapeutics, including GDMT for heart failure, anticoagulation, catheter ablation and device implantation for arrhythmias are likely to have a significant impact on the mortality of this disease. Patient-specific genetic assays are becoming more widely available and may help with future risk stratification or offer targets for gene therapies [91]. Prenatal testing, although not formally recommended, has been suggested as a means of early detection for congenital DM should mothers display high risk features, such as expansions >1 kb, or birth to another sibling with congenital DM [92].

As suggested by current society guidelines, there is no therapy yet to replace a high index of clinical suspicion for cardiac complications of DM by the treating physician. Regular health maintenance with an attention to cardiac symptoms is paramount in ensuring the safety and survival of patients with DM.
